# Risk factors and clinical features of deterioration in COVID-19 patients in Zhejiang, China: a single-centre, retrospective study

**DOI:** 10.1186/s12879-020-05682-4

**Published:** 2020-12-10

**Authors:** Ping Yi, Xiang Yang, Cheng Ding, Yanfei Chen, Kaijin Xu, Qing Ni, Hong Zhao, Yongtao Li, Xuan Zhang, Jun Liu, Jifang Sheng, Lanjuan Li

**Affiliations:** 1 State Key Laboratory for Diagnosis and Treatment of Infectious Diseases, National Clinical Research Centre for Infectious Diseases, Collaborative Innovation Centre for Diagnosis and Treatment of Infectious Diseases, the First Affiliated Hospital, College of Medicine, Zhejiang University, Qingchun Road 79, Hangzhou, 31003 Zhejiang Province People’s Republic of China; 2 Department of Infectious Disease, ShuLan Hangzhou Hospital, Hangzhou, Zhejiang Province China

**Keywords:** Coronavirus disease 2019 (COVID-19), Severe acute respiratory syndrome coronavirus 2 (SARS-CoV-2), Predictors, Severe illnesses

## Abstract

**Background:**

Severe acute respiratory syndrome coronavirus 2 (SARS-CoV-2) infection swept through Wuhan and spread across China and overseas beginning in December 2019. To identify predictors associated with disease progression, we evaluated clinical risk factors for exacerbation of SARS-CoV-2 infection.

**Methods:**

A retrospective analysis was used for PCR-confirmed COVID-19 (coronavirus disease 2019)-diagnosed hospitalized cases between January 19, 2020, and February 19, 2020, in Zhejiang, China. We systematically analysed the clinical characteristics of the patients and predictors of clinical deterioration.

**Results:**

One hundred patients with COVID-19, with a median age of 54 years, were included. Among them, 49 patients (49%) had severe and critical disease. Age ([36–58] vs [51–70], *P* = 0.0001); sex (49% vs 77.6%, *P* = 0.0031); Body Mass Index (BMI) ([21.53–25.51] vs [23.28–27.01], *P* = 0.0339); hypertension (17.6% vs 57.1%, *P* < 0.0001); IL-6 ([6.42–30.46] vs [16.2–81.71], *P* = 0.0001); IL-10 ([2.16–5.82] vs [4.35–9.63], *P* < 0.0001); T lymphocyte count ([305–1178] vs [167.5–440], *P* = 0.0001); B lymphocyte count ([91–213] vs [54.5–163.5], *P* = 0.0001); white blood cell count ([3.9–7.6] vs [5.5–13.6], *P* = 0.0002); D2 dimer ([172–836] vs [408–953], *P* = 0.005), PCT ([0.03–0.07] vs [0.04–0.15], *P* = 0.0039); CRP ([3.8–27.9] vs [17.3–58.9], *P* < 0.0001); AST ([16, 29] vs [18, 42], *P* = 0.0484); artificial liver therapy (2% vs 16.3%, *P* = 0.0148); and glucocorticoid therapy (64.7% vs 98%, *P* < 0.0001) were associated with the severity of the disease. Age and weight were independent risk factors for disease severity.

**Conclusion:**

Deterioration among COVID-19-infected patients occurred rapidly after hospital admission. In our cohort, we found that multiple factors were associated with the severity of COVID19. Early detection and monitoring of these indicators may reduce the progression of the disease. Removing these factors may halt the progression of the disease. In addition, Oxygen support, early treatment with low doses of glucocorticoids and artificial liver therapy, when necessary, may help reduce mortality in critically ill patients.

**Supplementary Information:**

The online version contains supplementary material available at 10.1186/s12879-020-05682-4.

## Background

In December 2019, a new type of coronavirus disease (COVID-19) occurred in Wuhan, China, which was caused by a novel enveloped RNA betacoronavirus 2 [1]. Due to its phylogenetic similarity to severe acute respiratory syndrome coronavirus (SARS-CoV), COVID-19 was also named SARS-CoV-2 [2]. SARS-CoV-2 infection has become a public health emergency of international concern since it has rapidly spread within China and in several other countries. As of 1 pm 19 February 2020, a total of 74,280 cases of COVID-19 were confirmed in China, with a total of 2008 deaths [3]. The latest mortality was approximately 2.3% [4]. The infection is highly contagious and can be transmitted from person to person, leading to cluster transmission in families [5]. However, Clinical manifestations of SARS-CoV-2 range from asymptomatic to severe acute respiratory syndrome [1]. Due to these unknown factors, there is currently no specific treatment for SARS-CoV-2, and the risk of death for severely ill patients is very high. Early detection of severe cases can reduce mortality. In this paper, a retrospective analysis of inpatients diagnosed with SARS-CoV-2 in Hangzhou, Zhejiang, China, over the period from January 19, 2020, to February 19, 2020, was performed to explore the relevant factors that may predict the risk factors for disease severity.

## Method

### Study design and participants

We included in the study all patients diagnosed with SARS-CoV-2 who were admitted to the First Affiliated Hospital of Zhejiang University School of Medicine between January 19, 2020, and February 19, 2020. This study was approved by the ethics review committee of the First Affiliated Hospital of Zhejiang University and conforms to the code of ethics of the Helsinki declaration in 2013.

The degree of severity of SARS-CoV-2 (severe vs non-severe) at admission was assessed using the latest guidelines for SARS-CoV-2 diagnosis from the National Health Commission of China [6]. As outlined in the guidelines, patients were classified into four types: mild, moderate, severe, and critical illness. Mild cases included patients with mild clinical symptoms and no signs of pneumonia on imaging. Moderate cases included patients with fever, respiratory symptoms, and imaging manifestations of pneumonia. Severe cases included patients who met one of the following criteria: respiratory rate ≥ 30 breaths per minute; arterial oxygen saturation (SaO2) ≦ 93% at rest; or partial pressure of oxygen (PaO2)/ Fraction of Inspiration O2 (FiO2) ≦300 mmHg. Critical illness cases included patients who met any of the following criteria: respiratory failure requiring mechanical ventilation, occurrence of shock, and complications with other organ failure requiring care in an intensive care unit. Mild and moderate cases were categorized as non-severe cases, while severe and critical illness cases were defined as severe cases in this study.

### Cytokine and lymphocyte measurement

Plasma cytokines (IL6, IL10) were measured in all patients using the Human Cytokine standard 27-plex assay panel and the Bio-PLEX 200 system (Bio-RAD, Hercules, CA, USA) according to the manufacturer’s instructions. Lymphocyte subset counts in peripheral blood [CD45+, CD3+ CD4+, CD3-CD19+ and CD3-CD16+ CD56+]were measured by flow cytometry in accordance with the manufacturer’s protocol. (BD FACS Calibur, USA). They were completed by the laboratory department of our hospital and the State Key Laboratory of Diagnosis and Treatment of infectious diseases.

### Data collection

Data were collected from electronic medical records for the following parameters: demographics, underlying medical comorbidities, clinical symptoms and signs, laboratory tests, treatment. Laboratory tests consisted of IL-6, IL-10, lymphocyte subset count, white blood cell count, D2 dimer, PCT, CRP, and AST. Underlying medical comorbidities mainly included hypertension, diabetes and coronary heart disease. Treatment included antiviral, ambroxol, glucocorticoid, and artificial liver therapy.

### SARS-CoV-2 RT-PCR test

Respiratory tract samples, such as nasopharyngeal, sputum, or throat swab samples, from all included patients were collected daily (Nasopharyngeal or throat swab sampling is less than 10% of all samples). Using SARS-CoV-2 nucleic acid detection kits (Shanghai Biogerm Medical Technology Co Ltd), we detected SARS-CoV-2 viral RNA by polymerase chain reaction analysis in accordance with the manufacturer’s protocol. The diagnosis was confirmed by the criteria recommended by the National Institute for Viral Disease Control and Prevention (China). A viral cycle threshold (CT) value less than 37 was defined as positive, and a CT value greater than 40 was defined as negative. A CT value between 37 and 40 was confirmed by retesting.

### Statistics analyses

Continuous variables are presented as the median (Interquartile range IQR) and were compared by the Kruskal-Wallis test between the non-severe group and the severe group. Categorical variables were presented as numbers (%) and compared by the chi-square (χ^2^) test or Fisher’s exact test. The Kaplan-Meier (product-limit) method was used to evaluate the time periods from glucocorticoid treatment and artificial liver therapy to different clinical outcomes between the two groups. All statistical analyses were performed with SAS 9.4 software (SAS Institute Inc., Cary, NC, USA). The significance level of the hypotheses tests was set at 0.05 (two-sided).

### Patient and public participation

This was a retrospective cohort study in which the patients were not involved in the design of the study, the setting of the study questions, or the direct measurement of the results.

## Result

### Patient characteristics on admission

Of the 100 hospitalized SARS-CoV-2 patients between January 19, 2020, and February 19, 2020, 49 (49%) patients were critically ill. The demographic and clinical characteristics are shown in Table 1. The median age was 54 years. The age (median) of the severe group was significantly higher than that of the non-severe group (61 [51–70] vs 50 [36–58]; *P* = 0.0001]). Sixty-three percent of the patients were older than 50 years of age. A total of 63% of patients were male. In the severe group, the proportion of males was significantly higher than that of females (77.6% vs 49%; *P* = 0.0031). Overweight (BMI > 24) patients were common in both groups of COVID-19 patients. BMI was significantly higher in the severely ill group (24.44 (23.28–27.01) vs 24 (21.53–25.51); *P* = 0.0339). Underlying medical conditions such as hypertension (37%) and diabetes mellitus (11%) were the most common coexisting illnesses among SARS-CoV-2 patients. Moreover, the presence of hypertension was more common among patients with severe disease (57.1% vs 17.6; *P* < 0.0001). Fever was present in 71% of our patients, and there was no significant difference in fever between the two groups. In our study, the vast majority of patients (98%) had obvious pulmonary CT lesions. Especially in severe patients, pulmonary computed tomography (CT) lesions of different degrees were observed on admission, including patchy, nodular ground glass shadows and interstitial abnormalities.

**Table 1:** Clinical Characteristics of Patients With SARS-CoV-2 Infection

Characteristic	Total (*N* = 100)	Non-Severity (*n* = 51)	Severity (*n* = 49)	*P* Value*
Demographics, n (%)				
Age, median (IQR), y	54 (42–64)	50 (36–58)	61 (51–70)	0.0001
≥ 50	63 (63)	26 (51)	37 (75.5)	0.0111
Male sex	63 (63)	25 (49)	38 (77.6)	0.0031
BMI	24.27 (22.14–25.97)	24.01 (21.53–25.51)	24.44 (23.28–27.01)	0.0339
Mainly underlying conditions, n (%)				
Hypertension	37 (37)	9 (17.6)	28 (57.1)	< 0.0001
Diabetes mellitus	11 (11)	3 (5.9)	8 (16.3)	0.3574
Cardiac disease	4 (4)	1 (2)	3 (6)	0.118
Fever on admission-°C				
< 37.5 °C	29 (29)	19 (37.3)	10 (20.4)	0.0635
Fever during hospitalization				
Median highest temperature (IQR)	38.25 (37.5–39)	38.2 (37.6–39)	38.3 (37.5–39)	0.9669
Radiologic findings on admission				
chest CT — no./total no. (%)				
Normal	2 (2)	2 (3.9)	0	0.4952
Initial laboratory findings, median (IQR)				
WBC, 10^9^/L	6.4 (4.1–10.8)	5.15 (3.9–7.6)	9.1 (5.5–13.6)	0.0002
d-dimer	452.5 (240–857.5)	339 (172–836)	604 (408–953)	0.005
ALT	22 (15–40.5)	20 (13–41)	24 (15–40)	0.2801
AST	20.5 (16–34)	20 (16–29)	25 (18–42)	0.0484
CRP	24.665 (7.785–47.6)	10.9 (3.8–27.9)	39.78 (17.3–58.9)	0.0001
PCT	0.06 (0.03–0.1)	0.04 (0.03–0.07)	0.07 (0.04–0.15)	0.0039
IL2	0.95 (0.855–1.82)	0.95 (0.78–1.83)	0.95 (0.95–1.81)	0.5534
IL6	20.425 (8.78–56.04)	12.52 (6.42–30.46)	38.22 (16.2–81.71)	0.0001
IL10	4.775 (2.91–8.03)	3.45 (2.16–5.82)	6.93 (4.35–9.63)	< 0.0001
TNF-a	17.98 (10.945–59.085)	23.29 (12.2–65.49)	12.22 (10.77–36.44)	0.1658
IFN-r	10.06 (5.12–33.56)	11.4 (4.92–39.71)	9.82 (5.19–28.57)	0.6943
ALC	703 (402–1119)	1119 (620–1554)	543.5 (367.5–810.5)	0.0002
Total T cells	348 (233–775)	752 (305–1178)	276.5 (167.5–440)	0.0001
Th cells	176 (79–431)	376 (176–618)	115 (60.5–214.5)	< 0.0001
B cells	115 (70–211)	167 (91–213)	93.5 (54.5–163.5)	0.0189
NK cells	105 (64–183)	154 (70–207)	101 (61.5–155.5)	0.1201

*Abbreviation*: *IQR* Inter quartile range, *BMI* Body mass index, *WBC* White blood count, *ALT* Aspartate aminotransferase, *AST* Alanine aminotransferase, *CRP* C-reactive protein, *PCT* Procalcitonin, *IL* Interleukin, *TNF-a* Tumor necrosis factor a, *IFN* Interferon, *Th* T helper cells, *ALC* Absolute lymphocyte count, *NK cells* Natural killer cells

*, Chi-square (χ^2^) test or Fisher’s exact test was used with *P* < 0.05 as significant

### Laboratory tests

Among laboratory indicators at admission, the white blood cell count ([3.9–7.6] vs [5.5–13.6], *P* = 0.0002), D-dimer ([172–836] vs [408–953], *P* = 0. 005), C-reactive protein ([3.8–27.9] vs [17.3–58.9], *P* < 0.0001), procalcitonin ([0.03–0.07] vs [0.04–0.15], *P* = 0.0039) and alanine aminotransferase ([16, 29] vs [18, 42], *P* < 0.0484) in the non-severe group were significantly lower than those in the severe group. In our study, we mainly focused on the laboratory detection of cytokines and immune cell subsets in patients with SARS-CoV-2 infection. We found that the expression of IL-6 in severe patients was significantly higher than that in the non-severe group ([6.42–30.46] vs [16.2–81.71], *P* = 0.0001), while the expression of IL-10 in severe patients was significantly lower than that in the non-severe group ([2.16–5.82] vs [4.35–9.63], *P* < 0.0001). The total number of T cells ([305–1178] vs [167.5–440], *P* < 0.0001), B cells ([91–213] vs [54.5–163.5], *P* = 0.0189), absolute number of lymphocytes ([620–1554] vs [367.5–810.5], *P* = 0.0002), Th ([176–618] vs [60.5–214.5], *P* < 0.0001) and Ts/Tc cells ([137–443)] vs [69–140.5], *P* < 0.0001) were significantly lower than those in non-severe cases.

### Treatment and clinical outcomes

The treatment and clinical outcomes of COVID-19 patients are shown in Table 2. All 100 patients received antiviral treatment including lopinavir/ritonavir, interferon-α, darunavir/cobicistat, favipiravir and arbidol. The median time from symptom onset to antiviral regimen administration was 7 (4–9.5) days, with no significant difference between the non-severe and severe groups. The median time from antiviral regimen administration to negative results for SARS-CoV-2 RNA for the first time was 9 (5–14) days. Moreover, the time in the non-severe group was significantly shorter than that in the severe group (6 (4–12) vs 9 (7–15) days; *P* = 0.0142). The median duration from antiviral treatment to the time of a negative viral testing result was 10 (6–15) days, and the duration of the non-severe group was significantly shorter than that of the severe group (7 (4–13) vs 12 (9–18); *P* = 0.0006). In addition, our patients received supportive symptomatic treatments, including oxygen, glucocorticoid, ambroxol, antibiotic, and artificial liver therapy. Glucocorticoids were given to 80 (80%) patients, and the proportion was significantly higher in the severe group than in the non-severe group (98% vs 64.7%; *P* < 0.0001). Meanwhile, the maximum dosage of glucocorticoids in the severe group was significantly higher than that in the non-severe group (40[40–80] vs 40 [0–40]; *P* = 0.0001). Compared with the non-severe group, the severe group had a higher rate of antibiotic treatment (44.9% vs 3.9%; *P* < .0001). In the retrospective analysis, we found that the clinical outcome of our patients was very good. No patients died, and 86% of the patients recovered and were discharged from the hospital. The discharge rate of the non-severe group was significantly higher than that of the severe group (96.1% vs 75.5%; *P* = 0.003). The Intensive Care Unit (ICU) admission rate was 23%, which was higher in the severe group (42.9% vs 3.9%; *P* < 0.0001).

**Table 2:** Comparison of treatment responses and clinical outcomes of patients infected with SARS-CoV-2 between non-severe and severe group

Variable	Total (***N*** = 100)	Non-Severity (***n*** = 51)	Severity (***n*** = 49)	***P*** Value
**Treatments** n (%)				
**Antivirus treatment**	100 (100)	51 (100)	49 (100)	
median (IQR), days				
Time from illness onset to Antivirus start	7 (4–9.5)	6 (3–9)	7 (5–10)	0.2948
Glucocorticoid treatment n (%)	81 (81)	33 (64.7)	48 (98)	< 0.0001
Maximum dosage	80 (20–80)	80 (0–40)	80 (40–80)	0.0001
(equivalent methylprednisolone), median (IQR), mg/d				
Artificial liver support n (%)	9 (9)	1 (2)	8 (16.3)	0.0148
Antibiotic treatment n (%)	24 (24)	2 (3.9)	22 (44.9)	< 0.0001
Oxygen support				< 0.0001
Nasal cannular	64 (64)	46 (90.2)	18 (36.7)	
high-flow nasal cannula	13 (13)	3 (5.9)	10 (20.4)	
Invasive mechanical ventilation	23 (23)	2 (3.9)	21 (42.9)	
median (IQR), days				
^Α^AT to first virologic conversion	9 (5–14)	6 (4–12)	9 (7–15)	0.0142
AT to stable virologic conversion	10 (6–15)	7 (4–13)	12 (9–18)	0.0006
AT to radiologic recovery	7 (4–10)	7 (5–11)	6.5 (4–9.5)	0.3162
AT to temperature recovery	5 (2–8)	3 (2–7)	6.5 (2–9)	0.0903
**Clinical outcomes, n (%)**				
Discharge from hospital	86 (86)	49 (96.1)	37 (75.5)	0.0030
^Β^ICU admission	23 (23)	2 (3.9)	21 (42.9)	< 0.0001
Death	0	0	0	0

*Abbreviation*: ^Α^*AT* Antiviral therapy onset, ^Β^*ICU* Intensive care unit

### Risk factors for SARS-CoV-2 severe illness

Using a multivariate logistic regression analysis, we identified the risk factors associated with exacerbation of SARS-CoV-2 (Table 3). Age and BMI were recognized as predictors (independent factors) of severe illness. However, sex, hypertension, IL-6, T lymphocyte count, B lymphocyte count, glucocorticoid treatment and artificial liver support were not recognized as independent factors.

**Table 3:** Multivariate Logistic Regression analysis of risk factor for disease severity among hospitalized patients with COVID19

Variable	Odds Ratio (95% Confidence Interval)	***P*** Value
Age	1.064 (1.007–1.124)	**.027**
BMI	1.240 (1.006–1.528)	**.044**
IL6	1.005 (0.995–1.015)	.307
T cells	1.003 (0.995–1.011)	.424
B cells	1.005 (0.997–1.014)	.196
Artificial liver support	0.985 (0.073–13.211)	.99

## Discussion

In December 2019, a new infectious disease, SARS-CoV-2, swept through Wuhan and quickly spread to all Chinese cities and dozens of countries overseas. Similar diseases are highly infectious and can spread through human-to-human transmission among close contacts and over a wide area [7, 8]. However, the source of SARS-CoV-2 is not fully clear, and the lack of specific treatments may cause the patient’s symptoms to progress from mild to severe or even result in death. Previous studies reported that the fatality rate among hospitalized SARS-CoV-2 patients was approximately 2.3%, which was much lower than those of SARS- and Middle East Respiratory Syndrome (MERS)-infected patients [4, 9]. For such patients, we need to remain vigilant and provide symptomatic supportive treatment early to reduce the occurrence of severe disease.

In our retrospective cohort study, we included 100 SARS-CoV-2 patients from a single clinical centre in Hangzhou, Zhejiang Province. Our results showed that age and BMI were independent risk factors for severe illness. The median patient age was 54 years. Sixty-three percent of the patients were older than 50 years, which was consistent with multiple reports in the literature [10, 11]. Severe patients were much older than non-severe patients, which may be related to a lower immune response and a higher frequency of underlying conditions that are not beneficial for self-limited recovery after virus infection. In addition, we discovered that overweight (BMI > 24) was an important risk factor for severity in SARS-CoV-2 patients. Compared with the BMI of severe patients, non-severe patients had a lower BMI, with significant differences between the two groups (*P* = 0.0339).

Similar to H7N9, the most common symptoms of SARS-CoV-2 patients were fever and cough [5, 12]. However, 26% of SARS-CoV-2 patients who were admitted to our hospital had a normal temperature. If these patients had been overlooked as having SARS-CoV-2, then more people might have been infected. This was similar to previous reports [13]. Coexisting disorders such as hypertension and diabetes mellitus were associated with severe illnesses. The proportions of hypertension and diabetes mellitus in severe illnesses were higher than those in non-severe cases. Furthermore, hypertension was significantly different between the two groups (*P* < 0.0001). This was consistent with H7N9 patients [14]. In addition, we also found that the laboratory parameters and supplementary treatment, including the white blood cell count, D-dimer, C-reactive protein, procalcitonin, alanine aminotransferase, IL-6, IL-10, CD3+ *T*-cell, CD4+ *T*-cell (CD3 + CD4+), CD8+ *T*-cell (CD3 + CD8+), B-cell (CD3 − CD19+) and NK-cell (CD3 − CD16 + CD56+), absolute lymphocyte number, and TS/Tc cells and the need for antibiotic, glucocorticoid and artificial liver therapy, were identified in single variable analysis as risk factors for severe illnesses but that these risk factors did not reach our criteria for significance in the multivariate analysis.

Previous studies found that an inflammatory factor storm was one of the important factors for severe illness and even death of H7N9, SARS-CoV, MERS-CoV. The mechanism may be related to the overexpression of inflammatory factors and chemokines, which can lead to acute lung injury and ARDS [1, 15, 16]. In our retrospective study, we also found a significant increase in cytokine IL-6 in the patients with severe disease. Therefore, for patients with a gradual increase in the expression of such inflammatory factors, early low-dose glucocorticoid and artificial liver treatment may alleviate the progression of the disease and reduce the risk of death. The effectiveness and necessity of glucocorticoid use has been controversial in novel coronavirus infections. Large doses of glucocorticoids may cause significant side effects such as lymphocytopenia and femoral head necrosis [17]. In our cohort, the rates of treatments with glucocorticoids and artificial liver therapy in the severe group were greater than those in the non-severe group, and there were significant differences. However, there were no statistically significant differences in the treatment effects between the two groups (the days from treatment initiation to virus clearance) (supplementary figures 1–2), which may be related to our small sample size. Further research on larger cohorts is in progress. Our study found that the number of immune cells in most patients with mild disease is normal. The decrease in T lymphocytes, B lymphocytes and absolute lymphocyte counts are correlated with the severity of the disease. Therefore, early detection of related immune indicators may provide us with the ability to predict the severity of COVID19.

There are several limitations to be considered when interpreting the findings. First, our study is a single-centre study of SARS-CoV-2 risk factors for critically ill patients in a hospital. Furthermore, when screening confirmed cases, the vast majority of tested samples were from lower respiratory tract specimens, but there were still a few pharyngeal swab specimens, and the pharyngeal swab false negative rate may have led to the lack of included patients. Finally, the number of children under 18 years in our sample was small, so no specific conclusions could be drawn for adolescents.

## Conclusions

Patients with SARS-CoV-2 infection are likely to progress to critical illness. In our analysis, we found that pre-existing diseases and multiple laboratory indicators were associated with disease progression. Age and BMI may be independent risk factors for the development of severe SARS-CoV-2. Therefore, it is important to evaluate the severity of the newly diagnosed patients’ condition to provide individualized diagnosis and treatment and to improve the prognosis.

## Supplementary Information

**Additional file 1.**

## Data Availability

The datasets used and/or analyzed during the current study are available from the corresponding author and data was publicly available on reasonable request.

## Abbreviations

COVID-19: Coronavirus Disease 2019
SARS-CoV-2: Severe Acute Respiratory Syndrome Coronavirus 2
SaO2: Arterial Oxygen Saturation
PaO2: Partial Pressure of Oxygen
FiO2: Fraction of Inspiration O2
CT value: Cycle Threshold Value
IQR: Interquartile range
BMI: Body Mass Index
CT: Computed Tomography
ICU: Intensive Care Unit
