# Supplementary material for: Risk factors and clinical features of deterioration in COVID-19 patients in Zhejiang, China: a single-centre, retrospective study
Source: BMC Infect Dis. 2020 Dec 10;20:943. doi: 10.1186/s12879-020-05682-4 (PMC7726595; doi:10.1186/s12879-020-05682-4)
Supplement: Supplementary file 1 — Additional file 1. [file 12879_2020_5682_MOESM1_ESM.docx]

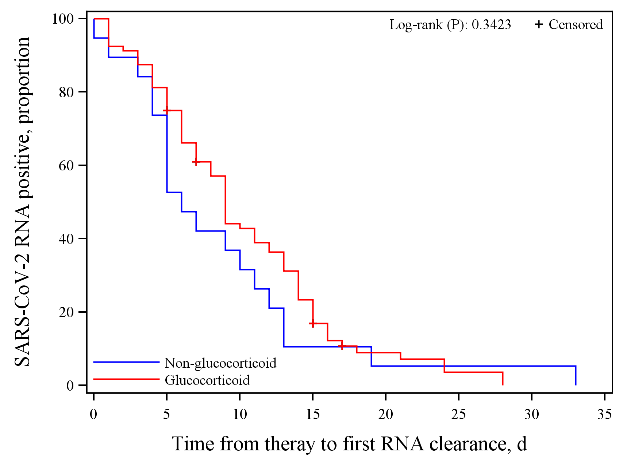

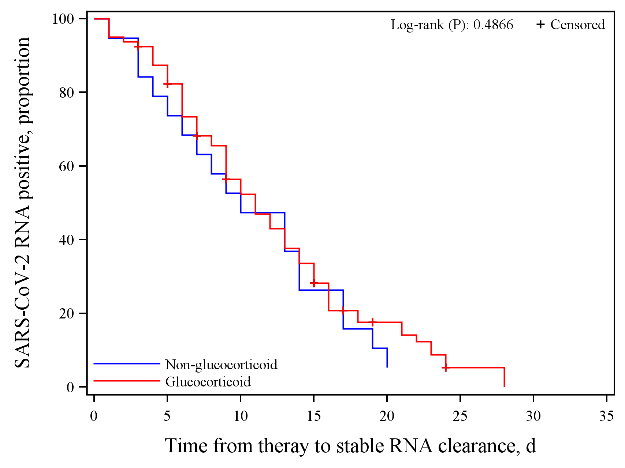


B

A

**Supplementary Figure 1. Kaplan-Meier analysis of treatment responses between groups with** **glucocorticoid.** Glucocorticoid had no significant effects on the virologic conversion with nucleic acid RT-PCR turning from positive to negative **(A)** nor the duration of sustained negative results **(B).**


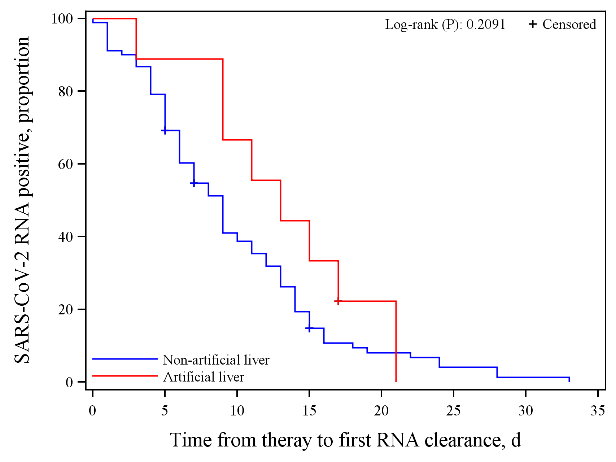

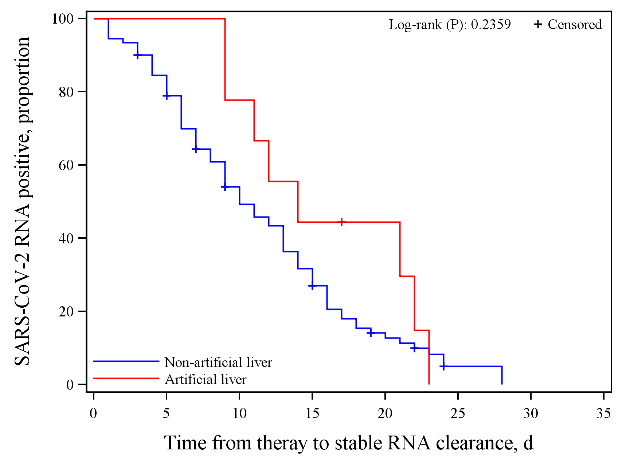


B

A

**Supplementary Figure 2. Kaplan-Meier analysis of treatment responses between groups with artificial liver therapy.** Artificial liver therapy had no significant effects on the virologic conversion with nucleic acid RT-PCR turning from positive to negative **(A)** nor the duration of sustained negative results **(B)**.
